# Effect of Calcitriol on the Renal Microvasculature Differentiation Disturbances Induced by AT_1_ Blockade During Nephrogenesis in Rats

**DOI:** 10.3389/fmed.2020.00023

**Published:** 2020-02-06

**Authors:** Amanda L. Deluque, Lucas F. de Almeida, Heloísa D. C. Francescato, Cleonice G. A. da Silva, Roberto S. Costa, José Antunes-Rodrigues, Terezila M. Coimbra

**Affiliations:** ^1^Laboratory of Renal Physiology, Department of Physiology, Ribeirão Preto Medical School, University of São Paulo, São Paulo, Brazil; ^2^Laboratory of Renal Pathology, Division of Nephrology, Department of Internal Medicine, Ribeirão Preto Medical School, University of São Paulo, São Paulo, Brazil; ^3^Laboratory of Neuroendocrinology, Department of Physiology, Ribeirão Preto Medical School, University of São Paulo, São Paulo, Brazil

**Keywords:** calcitriol, cell differentiation, kidney development, renin-angiotensin system, renal microvasculature

## Abstract

Alterations in the renal vasculature during fetal programming can cause disturbances in renal structure and function that persist into adulthood. Calcitriol can affect cellular differentiation and proliferation, and promote endothelial cell maintenance, each of which is a key event in nephrogenesis. Calcitriol is a negative endocrine regulator of the renin gene. Rats exposed to renin-angiotensin system (RAS) antagonists during lactation have been shown to develop renal disorders, which demonstrated that the RAS may play an important role in mammalian kidney development. We evaluated the effects of calcitriol administration on losartan [angiotensin II receptor antagonist (ANGII), AT_1_]-induced changes in renal differentiation in rats during lactation. Rats treated with losartan showed alterations in renal function and structure that persisted into adulthood. These disruptions included hydronephrosis, papillary atrophy, endothelial dysfunction, and aberrant endothelial structure. These changes were mitigated by treatment with calcitriol. The results of our study showed that animals exposed to AT_1_ blockade during lactation exhibited altered renal microvasculature differentiation in adulthood that was attenuated by treatment with calcitriol.

## Introduction

During renal development, a series of events occur that result in the formation of blood vessels (1). These processes include formation of the renal arterial tree and glomerular capillaries, and alignment of the vasa recta and peritubular capillaries (2). Three-dimensional organization and expansion of microvessels in the renal medulla are dependent on several signaling pathways that supply oxygen and nutrients to adjacent cells (2, 3). The renin-angiotensin system (RAS) plays an important role in expansion, migration, and formation of renal structure during nephrogenesis (4). Results from our studies and others have shown that pharmacological RAS inhibition or genetic deletion of downstream signaling components of RAS (1, 3, 5–8) result in impaired development of glomeruli and preglomerular vasculature. Angiotensin II (ANGII) also affects the post-glomerular circulation. A study performed by Madsen et al. showed that treatment with an AT_1_ receptor antagonist for 14 days during lactation reduced the volume, length, and surface area of capillaries in the kidney medulla, and resulted in disorganization of vasa recta bundles (3). In humans, nephrogenesis is complete by 34–36 weeks of gestation. In contrast, nephrogenesis continues after birth for about 2–3 weeks in mice and rats (1, 8). Therefore, neonatal rats are commonly used to study the mechanisms of renal development (5).

Several studies have reported that calcitriol (1α,25-dihydroxyvitamin D3; activated vitamin D) is involved in maintenance of endothelial function (9), cell proliferation, differentiation, apoptosis, and RAS modulation (5, 10, 11). We previously reported that treatment with calcitriol attenuated glomerular disturbances and promoted differentiation of tubular cells in the renal cortex in animals exposed to AT_1_ blockade during nephrogenesis (5). Blockade of AT_1_ with losartan during renal development resulted in overexpression of the renin gene and increased expression of ANGII (5), which resulted in disruption of nephrogenesis. Therefore, use of calcitriol in adulthood may mitigate losartan-induced effects on nephrogenesis through modulation of RAS. In addition, calcitriol may improve endothelial cell differentiation and function.

In the present study, we hypothesize that blockade of AT_1_ during renal development can result in disturbances in cell differentiation, resulting in reduced vascular density, and that treatment with calcitriol can mitigate these changes. Calcitriol has received increased attention because of widespread vitamin D deficiency and the recent discovery that calcitriol can modulate the RAS. Further evaluation of the effects of calcitriol is important because losartan is a typical pharmacological treatment for hypertension, even in pregnancy. Therefore, the aim of our study was to evaluate the effects of calcitriol on losartan-induced disturbances in the renal microvasculature. Capillary density was evaluated in kidney tissue by immunohistochemical staining for aminopeptidase P (JG12). Associations between capillary density and parenchymal changes in the outer and inner medulla were analyzed using markers of cell differentiation (vimentin and α-SMA).

## Materials and Methods

### Animals and Experimental Design

All experiments were performed in accordance with the ethical principles for animal experimentation of the Brazilian College of Animal Experimentation, and the Animal Experimentation Committee of the University of São Paulo at Ribeirão Preto Medical School approved the study protocol (COBEA/CETEA/FMRP-USP, protocol no. 178/2014).

The animals were housed in a controlled temperature (22°C) environment and exposed to a 12 h light/12 h dark cycle. The animals were provided chow diet and water *ad libitum*. Female and male Wistar rats (Animal House of the Campus of Ribeirao Preto, University of São Paulo, Ribeirao Preto, SP, Brazil) were housed together overnight to permit mating. Each male was housed with three females, and the first gestational day was determined based on the presence of copulatory plugs (12). Pregnant females were separated and monitored during of the entire gestation period and during lactation (21 days each). Male Wistar pups were randomly selected for this study. The litter was reduced to six per mother to ensure equal feeding. During lactation, rats were divided into the following groups: (1) offspring of mothers treated with 2% sucrose solution during lactation and (2) offspring of mothers treated with losartan during lactation (100 mg/kg/day; All Chemistry, Brazil) (5, 7). Each group received the treatment solutions in place of drinking water. Losartan was detected in breast milk at a concentration nearly half of that in maternal plasma in 75% of the rats (8).

After weaning, losartan or sucrose treatments were discontinued, and the animals were separated from their mothers and allowed to acclimate for 4 days. The rats were then separated into the following four experimental groups: (1) SUC (sucrose 2%, *n* = 7), (2) SUC + Calcitriol (sucrose 2% + calcitriol, *n* = 7), (3) LOS (losartan, *n* = 8), and (4) LOS + Calcitriol (losartan + calcitriol, *n* = 8). Calcitriol (6 ng/day, Calcijex, Abbott Laboratories, USA) or vehicle (0.9% NaCl) was administered using mini-osmotic pumps (Model 2004, Alzet, USA) implanted subcutaneously under anesthesia with isoflurane (Cristalia, Brazil). Calcitriol or vehicle supplementation was started following the end of nephrogenesis when losartan-induced lesions were established and continued for 4 weeks. The dose and duration of calcitriol treatment were selected according to previous studies (5, 13, 14).

### Systolic Blood Pressure

Systolic blood pressure (SBP) was determined indirectly at 60 days of age using the tail—cuff method (CODA Non-Invasive Blood Pressure System, Kent Scientific Corporation, 2010). The animals were allowed to acclimate for 3 days prior to measurement of SBP. Twelve SBP measurements were averaged for each animal (15).

### Evaluation of Renal Function

At 59 days of age, the animals were placed in metabolic cages for 24 h to collect urine samples for measurement of sodium (9180-electrolyte analyzer, Roche, Austria) and osmolality (Fiske OS Osmometer, Advanced Instruments, USA). On the next day, the rats were weighed, then anesthetized using sodium thiopental (0.1 ml/100 g, Brazil). Blood samples were collected from the abdominal artery for analysis of creatinine (Labtest Diagnostica, Brazil) and sodium. One kidney was removed and fixed using methacarn solution for histological and immunohistochemical analyses.

### Determination of Nitric Oxide in Renal Tissue

Renal tissue was homogenized in 0.1 N acetic acid (3:1), centrifuged at 10,000 × g for 5 min, and aliquoted. The samples were deproteinated by addition of 95% ethanol (4°C) (1:2), then centrifuged at 4,000 × g for 5 min. The supernatants were analyzed for nitric oxide (NO) content by an NO/ozone technique described previously (16) using a Sievers analyzer (Sievers 280 NOA, USA). Protein levels in renal tissue were also determined as described previously (17).

### Histological Analysis

**Figure 1:**
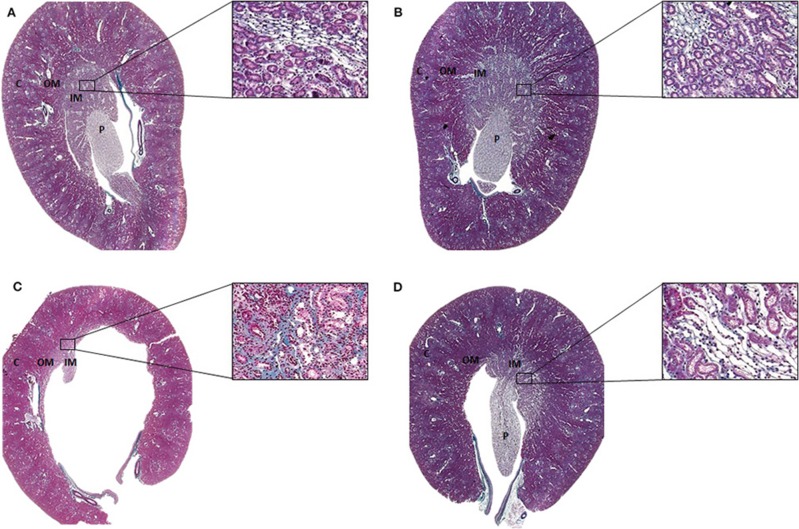
Representative Masson's trichrome staining of histological sections of the **(A)** SUC, **(B)** SUC + Calcitriol, **(C)** LOS, and **(D)** LOS + Calcitriol groups. C, Renal cortex; OM, Outer medulla; IM, Inner medulla; P, Papilla. Magnification, 1X and 40X.

Tissues were embedded in paraffin and sliced into 4-μm-thick slices, then stained with Masson's Trichrome and visualized using a light microscope (AxioVision Rel. 4.3; Zeiss, Germany). The outer and inner medulla were identified by location and epithelial characteristics. The transition from the cortical region to the medullary region was observed. A representative image is presented in Figure 1.

### Immunohistochemical Analysis

Kidney sections were deparaffinized and hydrated for immunohistochemical analysis. Non-specific antigen binding was blocked by incubation for 20 min with normal goat serum. The sections were then incubated with anti-vimentin (1:500, Dako Corporation M0725, Denmark), anti-aminopeptidase P (JG12, 1:1000, eBioScience BMS1104, USA), or anti-eNOS (1:100, Santa Cruz Biotechnology sc-376751, USA) antibodies for 60 min at room temperature, and anti-α-smooth muscle actin (α-SMA, 1:1000, Dako Corporation M0851, Denmark) antibody overnight at 4°C. Avidin-biotin-peroxidase complex (Vector Laboratories, USA) and DAB [3,3′-diaminobenzidine (Sigma Chemical Company, USA)] were used for detection. The sections were then counterstained with methyl green, dehydrated, and mounted.

The outer and inner medulla were evaluated. The images were randomly quantified using a Greek box system. Quantification was performed by a blinded analyst. The number of JG12-positive capillaries was counted and localization of α-SMA, vimentin, and eNOS was semi-quantitatively graded as follows: 0 = absent or <5% staining; 1 = 5–25% staining; 2 = 25–50% staining; 3 = 50–75% staining, and 4 ≥75% staining (18). Thirty consecutive fields (0.1 mm^2^ each) were evaluated for the outer and inner medulla. Only the inner medulla is shown in the figures.

### Western Blot Analysis

Kidneys were homogenized in lysis buffer (50 mM Tris–HCl, pH 7.4; 150 mM NaCl; 1% Triton X-100; 0.1% SDS; 1 μg/mL aprotinin; 1 μg/mL leupeptin; 1 mM phenylmethylsulfonyl fluoride; 1 mM sodium orthovanadate, pH 10; 1 mM sodium pyrophosphate; 25 mM sodium fluoride; and 0.001 M EDTA, pH 8), then centrifuged at 4°C for 15 min at 10,000 rpm. Proteins (60 μg) were separated by polyacrylamide gel electrophoresis, transferred to nitrocellulose membranes, incubated for 1 h in blocking buffer (TBS, 5% skim milk), washed with TBS-T (TBS, 0.1% Tween 20, pH 7.6), then incubated with anti-AT_1_ (1:500, Santa Cruz Biotechnology, USA) antibody overnight at 4°C. Membranes were incubated with anti-GAPDH monoclonal antibody (1:1000; Sigma Chemical Co, USA) overnight at 4°C as a loading control. The membranes were then washed and incubated with horseradish peroxidase-conjugated goat anti-mouse (1:5000; Dako, Denmark) antibody for 1 h at room temperature. The membrane-bound antibodies were detected using SuperSignal West Pico Chemiluminescent Substrate (Pierce Chemical, USA), and the blots were visualized using an imaging system (Kodak Gel Logic 2200, USA). Band intensity was quantified by densitometry using ImageJ NIH image software (http://www.nih.gov) and was reported in arbitrary units. Protein quantitation was performed as previously described (17).

### Statistical Analysis

Nonparametric Kruskal—Wallis test followed by Dunn's post-test was used to analyze non-normally distributed data. Analysis of variance followed by the Newman—Keuls multiple comparisons test was used for analysis of normally distributed data. Statistical analyses were performed using GraphPad Prism version 7.0 for Windows (GraphPad Software, USA). The data were expressed as means ± standard error of the mean (S.E.M.). *P* < 0.05 were considered statistically significant.

## Results

### Renal Function Evaluation

Calcitriol treatment improved kidney dysfunction caused by AT_1_ receptor blockade during renal development. Rats treated with losartan had higher SBP than those in the SUC and SUC + Calcitriol groups. The rats in the LOS + Calcitriol group had higher SBP than the control groups but did not differ from the LOS group (Table 1). Body weight did not differ among the LOS (359.3 ± 9.5), SUC (381.6 ± 9.8), and SUC + Calcitriol (377.5 ± 3.5) groups at 60 days of age. However, body weight was higher in the LOS + Calcitriol group (399.7 ± 12.6) than that in the control group. Changes in water intake, urinary volume, fractional sodium excretion (${\text{FE}}_{\text{Na}}^{+}$), and GFR levels were less pronounced in the LOS + Calcitriol group than those in the LOS group (Table 1). Urinary osmolality (U) was lower in the LOS and LOS + Calcitriol groups compared to that in the control group. Calcitriol treatment did not affect this parameter (Table 1).

**Table 1:** Systolic blood pressure, twenty-four-hour water intake, urine volume, urinary osmolality (U), fractional excretion of sodium (${\text{FE}}_{\text{Na}}^{+}$), and GFR of 60-day-old pups in SUC, SUC + Calcitriol, LOS and LOS + Calcitriol groups.

	**SUC**	**SUC + Calcitriol**	**LOS**	**LOS + Calcitriol**
SBP (mmHg)	120.6 ± 6.3	122.5 ± 1.9	143.4 ± 1.8^**^^;^^*##*^	132.9 ± 1.9
Water intake (mL 100 g^−1^ 24 h^−1^)	54.6 ± 3.1	50.4 ± 1.9	120.8 ± 11.1^***^^;^^*###*^	88.9 ± 5.83^**^^;^^*##*^^;^ ^*$$*^
Urinary volume (mL 100 g^−1^ 24 h^−1^)	35.7 ± 2.5	35.3 ± 1.4	96 ± 7.5^**^^;^^*##*^	69.1 ± 4.6^*^^;^^#^
U_osm_(mOsm kg H_2_O^−1^)	1,475.0 ± 26.8	1,504.0 ± 24.8	632.4 ± 62.3^***^^;^^*###*^	754.4 ± 56.4^***^^;^^*###*^
GFR (ml min^−1^100 g^−1^)	0.81 ± 0.09	0.84 ± 0.06	0.60 ± 0.16^*^^;^^#^	0.71 ± 0.19^*^^;^^#^^;^ ^*$*^
${\text{FE}}_{\text{Na}}^{+}$ (%)	0.28 ± 0.04	0.33 ± 0.07	0.80 ± 0.05^***^^;^^*###*^	0.56 ± 0.04^***^^;^^*###*^^;^ ^*$$*^

Data are expressed as means ± SEM.

* P < 0.05 vs. SUC;

** P < 0.01 vs. SUC;

*** P < 0.001 vs. SUC;

# P < 0.05 vs. SUC + Calcitriol;

## P < 0.01 vs. SUC + Calcitriol;

### P < 0.001 vs. SUC + Calcitriol;

$ P < 0.05 vs. LOS;

$$ P < 0.01 vs. LOS.

SBP, systolic blood pressure.

*GFR, glomerular filtration rate*.

### Analysis of Renal Structure

The animals of the LOS group presented with renal alterations characterized by atrophic papilla, interstitial fibrosis, tubular atrophy, and tubular dilation. Calcitriol treatment reduced these structural changes induced by AT_1_ blockade during renal development (Figure 1).

### Immunohistochemistry Studies

**Figure 2:**
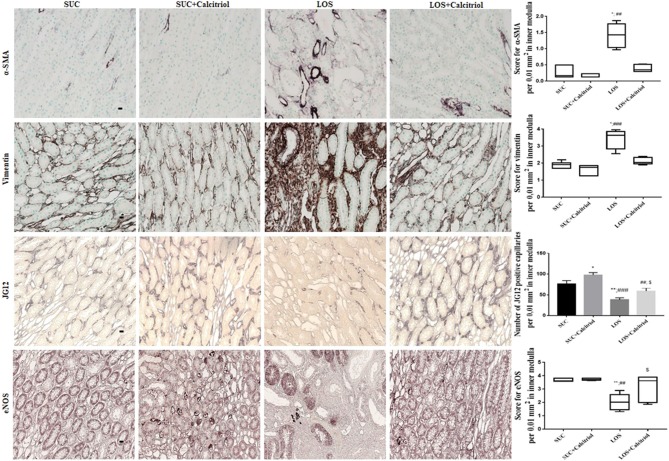
Immunolocalization of α-SMA, vimentin, JG12, and eNOS in inner medulla sections from the SUC, SUC + Calcitriol, LOS, and LOS + Calcitriol groups. Immunohistochemical data are expressed as the median and interquartile range (25–75th) and the mean ± S.E.M. Scale bar = 20 μm, *n* = 5–7 for each group. These markers indicated a lack of tubular and endothelial cell differentiation, and dysfunction. *P < 0.05 vs. SUC; **P < 0.01 vs. SUC; ^##^*P* < 0.01 vs. SUC + Calcitriol; ^###^*P* < 0.001 vs. SUC + Calcitriol; ^$^*P* < 0.05 vs. LOS.

The expression of α-SMA, a mesenchymal cell (myofibroblasts) marker, was increased in the outer medulla of the LOS group (1.23 ± 0.25) compared to that in the SUC (0.3 ± 0.03) and SUC+Calcitriol (0.26 ± 0.01) groups, which demonstrated lack of cellular differentiation in response to losartan. This increase was mitigated in the outer medulla of rats in the LOS + Calcitriol group (0.3 ± 0.04). Losartan also induced increased expression of α-SMA in the inner medulla, but treatment with calcitriol did not attenuate this increase (Figure 2).

The expression of vimentin, a marker of cell differentiation, was increased in the outer medullas of the LOS group (2.78 ± 0.31) compared to that in the SUC (1.05 ± 0.06) and SUC+Calcitriol (1.04 ± 0.07) groups. Losartan-induced increases in vimentin expression were mitigated by treatment with calcitriol (LOS+Calcitriol group; 1.08 ± 0.09). The expression of vimentin in inner medulla was higher in the LOS group than that in the SUC and SUC + Calcitriol groups. However, calcitriol treatment did not attenuate this increase (Figure 2).

Immunohistochemical analysis showed that the number of cells positive for JG12, an endothelial capillary cell marker, was reduced in the LOS group (20.73 ± 2.28) compared with that in the SUC (26.12 ± 2.89) and SUC+Calcitriol (23.88 ± 3.71) groups. These differences were not significant in the outer medulla. In the inner medulla, the number of endothelial cells was significantly lower in the LOS group than that in the SUC and SUC + Calcitriol groups. In addition, the LOS + Calcitriol exhibited significantly different expression of JG12 than the SUC + Calcitriol and LOS groups. Calcitriol treatment increased JG12 expression in the inner medullas of rats in the SUC + Calcitriol group compared with that in the SUC group (Figure 2).

Endothelial nitric oxide synthase (eNOS) expression was decreased in the kidneys of the LOS group (3.12 ± 0.24) compared with that in the SUC (3.80 ± 0.06) and SUC + Calcitriol (3.86 ± 0.02) groups. Calcitriol treatment did alter the expression of eNOS in the outer medulla. In contrast, in the inner medulla, the LOS + Calcitriol group exhibited higher eNOS expression than the LOS group. In addition, the levels of NO in renal tissue of rats in the LOS + Calcitriol group (7.71 ± 1.69 μM/μg protein) were higher than those in the SUC (4.21 ± 0.38 μM/μg protein), SUC + Calcitriol (2.97 ± 0.23 μM/μg protein), and LOS (5.59 ± 0.65 μM/μg protein) groups. These results showed that AT_1_ receptor blockade resulted in disruption of endothelial cell structure and function, and that calcitriol affected neovasculogenesis (Figure 2).

### Western Blot Studies

**Figure 3:**
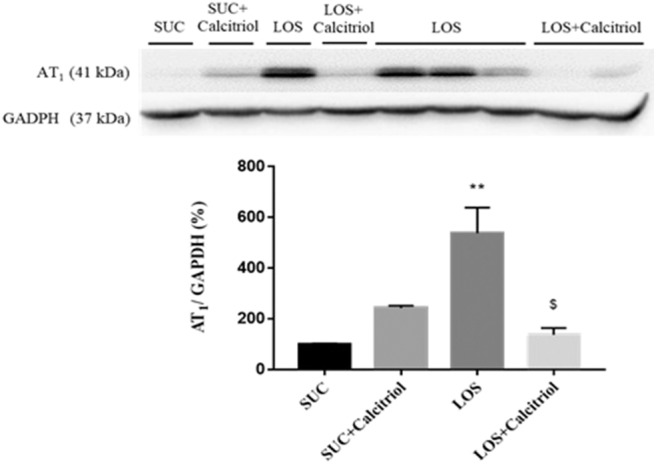
Intrarenal expression of AT_1_ receptor in the SUC, SUC+Calcitriol, LOS, and LOS + Calcitriol groups at 60 days of age. Immunoblot data are expressed as the mean ± S.E.M. **P < 0.01 vs. SUC; ^$^*P* < 0.05 vs. LOS.

The expression of AT_1_ was higher in the LOS group than that in the control groups and the LOS + Calcitriol group (Figure 3).

## Discussion

The results of this study showed that treatment with calcitriol attenuated disturbances in the renal microvasculature in adulthood induced by AT_1_-ANGII receptor inhibition during renal development. Several events that occur during development may contribute to hypertension in adulthood. Fetal programming results from mechanisms that include regulation of blood volume and blood pressure (1). Inhibition of AT_1_ in rats during renal development has been shown to result in increased blood pressure (8), reduced renal function, and altered renal structure in adulthood. These changes were associated with aberrant renal medulla microcirculation that resulted from losartan-induced reduction in urinary osmolality. Exposure to losartan reduced the ability to concentrate urine and increased urinary volume. This effect resulted from inner medulla and papillary atrophy-induced reductions in water reabsorption by the medullary collecting duct (19). These changes were exacerbated by decreased vasculature in the inner medulla, tubulointerstitial lesions, hydronephrosis, lack of differentiation of tubular cells, and formation of vascular medullar bundles, which resulted in disruption of the osmotic gradient. Expansion of renal medullary microcirculation during the embryonic stage in response to a properly functioning RAS (20) contributes to maintenance and control of sodium and water excretion (1). Reduced vessel density in the medulla resulting from renal development disorders that affect ANGII expression has been shown to induce changes in urinary concentration that could not be mitigated by calcitriol treatment. The integrity of vascular structures in the renal inner medulla is essential for urinary concentration (4), and is programmed in development, that could be dependent of arginine vasopressin (AVP), responsible to dipsogenic and pressor responses and to counter-current multiplier and exchange. The immature kidney from RAS inhibition to AVP may decrease water reabsorption in collecting duct and limit urine concentration. Impaired ANGII-AT_1_ receptor signaling or treatment with an AT_1_ receptor antagonist during kidney development results in reduced capillary density and impaired ability to concentrate urine that persists into adulthood. In our study, the urinary osmolality in the LOS + Calcitriol group was not significantly different from that in the LOS group, potentially because renal structure recovered and hydronephrosis induced by renal medulla loss was not complete. The absence of AT_1_ receptor signaling or loss of AT_1_ receptors can result in damage to ~50% of the renal parenchyma (3). We also observed an increase in ${\text{FE}}_{\text{Na}}^{+}$ in rats that received losartan during renal development, which indicated that renal sodium transport was altered. The changes in glomerular filtration rate observed in the present study were similar to those observed in other studies that used this model (5, 7, 20, 21).

Endothelial dysfunction has been shown to result in altered renal microvasculature with reduced peritubular capillary density (4). Serón et al. (22) and Bohle et al. (23) observed a negative correlation between the number of peritubular capillaries and renal function. We observed alterations in vascular bundles (Figure 1). Atrophic papilla and the presence of hydronephrosis and tubular dilation were characteristic of disruption of renal development by RAS blockade (4, 24). Calcitriol treatment attenuated these effects, likely through promotion of cell proliferation and differentiation. We previously showed that animals at 21 days postnatal exposed to losartan during lactation showed abnormalities in renal function, structure, hydronephrosis, and increased expression of mesenchymal markers (7, 20). Mecawi et al. (25) also observed hydronephrosis in animals exposed to captopril, an ACE (angiotensin-converting enzyme) inhibitor, characterized by distension and dilation of the renal pelvis. Based on these findings, hydronephrosis and alterations in medullar structure and function resulting from RAS inhibition were modulated, in part, by calcitriol. Morphometric changes have been previously evaluated only in the cortical region. The structures evaluated in previous studies included glomeruli, tubules, and cortical renal interstitium (5, 7, 20). As the RAS is important for renal development and AT_1_ receptor blockade induced rarefaction of renal vessels in the outer and inner medullas, the aim of our study was to evaluate changes in the medullary region and to correlate structural abnormalities with functional changes, and to evaluate the ability of calcitriol to mitigate these changes. In this study, we showed representative histological sections from kidneys of rats treated with sucrose or losartan and saline or calcitriol at lower magnification for visualization of papillary atrophy and hydronephrosis. All animals in the losartan group showed significant papillary atrophy, and treatment with calcitriol mitigated this atrophy. However, calcitriol treatment did not restore renal function in this model.

Alpha-smooth muscle actin is only present on arterioles and vessel walls in healthy animals (7, 26). In a ureteral obstruction model, calcitriol treatment reduced renal interstitial fibrosis and α-SMA expression, and preserved cell—cell interactions and epithelial phenotype, which resulted in suppression of fibrotic factors (27). The RAS is important in establishment of cell—cell interactions and interactions with the extracellular matrix (28). The lack of differentiation of medullary renal tubules induced by losartan was less pronounced in the group that received calcitriol, which showed that vitamin D was important in restoring cellular differentiation (5, 29, 30). Under normal conditions, vimentin, a component of the cytoskeleton, is present in glomerular epithelial cells and vessel walls (7). However, vimentin is only present in tubular cells in adulthood when differentiation, or cell proliferation, does not occur. Blockade of the AT_1_ receptor during renal development is associated with changes in α-SMA and vimentin expression in renal tissue, and results in lack of cell differentiation. Therefore, these proteins have been used as markers of impaired differentiation (5, 7, 20). Calcitriol treatment reduced vimentin expression in the outer medullas of the LOS group, and resulted in reduced mesenchymal cell expression. These results were consistent with those observed in other studies (5, 14, 31). These findings indicated that epithelial to mesenchymal transition (EMT) occurred in response to losartan, resulting in development of myofibroblasts. Tan et al. (27) showed that paricalcitol treatment reduced EMT, as evidenced by decreased mesenchymal markers in a model of obstructive nephropathy. Vitamin D acts by inhibiting the Snail transcription factor (involved in EMT programming) and restoring VDR (vitamin D receptor) expression.

We also showed that exposure to losartan resulted in development of renal vasculature disorders that were attenuated by calcitriol. We observed lack of differentiation of endothelial cells, as evidenced by reduced expression of JG12, a marker of blood vessel endothelial cells with greater specificity than the common endothelial markers CD34 and CD31, which are present in blood and lymphatic vessels (32). The reduced expression of eNOS in the renal medullas of animals exposed to losartan resulted in disturbances in endothelial function and structure that were attenuated by calcitriol treatment. These findings demonstrate that calcitriol could modulate cell differentiation and vascular function. Yoo et al. (4) reported decreased JG12 expression in rats exposed to an ACE inhibitor for 8 days after birth. Several clinical and experimental studies have shown that vitamin D can confer endothelial protection (5, 6, 9, 33, 34). A previous study showed that vitamin D deficiency could induce changes in peritubular capillaries, resulting in reduced capillary density (6). Several clinical and experimental studies have shown that vitamin D induces beneficial effects on endothelial cells (31). Use of vitamin D has also been shown to affect smooth muscle cell proliferation and positive impact on cardiovascular disease (31). These findings showed that vitamin D increased the number and function of endothelial cells. We observed increases in the expression of JG12 and eNOS in the inner medullas of animals treated with calcitriol in adulthood compared with animals treated with losartan alone. The effect of calcitriol on neovasculogenesis has not been characterized, but it has been shown to promote cell differentiation and proliferation, and to positively affect endothelial function and structure.

Rats in the LOS + Calcitriol group exhibited increased NO production. Nitric oxide is involved in endothelial vascular growth factor signaling. These angiogenic pathways are coordinated by ANGII during kidney development under normal physiological conditions and are activated by eNOS-induced Akt/PI3K signaling (35) and endothelial NO production. Phosphorylation of eNOS by Akt resulted in activation, and increased production of NO. (36). *In vitro* and *in vivo* studies have shown that increased eNOS activity resulted in endothelial cell proliferation and migration, and increased angiogenesis (37, 38). Increased NO production in the LOS + Calcitriol group compared to that in the LOS group have resulted in decreased vasoconstriction induced by reduced AT_1_ receptor activity in renal tissue from these animals. Decreased NO in the SUC + Calcitriol group may have been an artifact of small sample size. The expression of eNOS in the outer and inner medulla was equivalent between the SUC and SUC + Calcitriol groups. Previous studies have shown that vitamin D supplementation improved endothelial function in patients with chronic kidney disease (39). This effect was due to increased expression and activity of eNOS, which resulted in increased NO production (40, 41). Shear stress is reduced in chronic kidney disease due to reduced production of NO and growth factors such as VEGF and VEGFR2 (vascular endothelial growth factor and vascular endothelial growth factor receptor 2). Reduced shear stress results in induction of programmed cell death, resulting in a pro-inflammatory environment that contributes to reduced vascular density (42). Activated vitamin D can preserve the microvasculature and improve capillary blood flow and tissue perfusion (42), each of which were impaired following exposure to losartan during lactation. Recently, our group showed that vitamin D was important in maintenance of the renal microvasculature and played a key role in maintenance of homeostasis of growth factors such as VEGF and angiopoietins (angpt-1 and angpt-2), and the Tie-2 receptor (43).

The expression of the AT_1_ receptor was increased in the LOS group, likely due to a compensatory mechanism resulting from recovery of RAS activity. This is particularly interesting because exposure to losartan during lactation resulted in alterations that persisted into adulthood despite recovery of RAS signaling. This demonstrated that fetal programming was critical to development of hypertension. Calcitriol treatment mitigated LOS-induced increases in ANGII cortical expression (5). This resulted from increased AT_2_ receptor stimulation (21). Losartan treatment increased AT1 receptor expression which, after losartan withdrawal, reacted with increased cortical angiotensin II levels and that these effects were modulated by calcitriol. Blockade of AT_1_ disrupted the balance between proliferation and apoptosis, which resulted in altered renal structure. Calcitriol mitigated these microvasculature disturbances, which resulted in improved cell differentiation and function.

In conclusion, the present study showed that calcitriol treatment attenuated disturbances in endothelial function and inner medulla structure induced by losartan treatment during lactation. Calcitriol contributed to maintenance of the renal microvasculature in the outer and inner medullas of rats exposed to AT_1_ blockade during lactation, improved cellular differentiation, as evidenced by evaluation of α-SMA and vimentin. Calcitriol treatment also improved renal function and structure. Our study highlighted the importance of the renal medulla in development of proper kidney function.

## Data Availability Statement

All datasets generated for this study are included in the article/supplementary material.

## Ethics Statement

The animal study was reviewed and approved by Animal Experimentation Committee of the University of São Paulo at Ribeirão Preto Medical School approved the study protocol (COBEA/CETEA/FMRP-USP, protocol no. 178/2014).

## Author Contributions

AD, LA, HF, and CS contributed to the design of the experiments. The manuscript was written by AD and LA. TC supervised the study. RC performed histology and immunohistochemical staining. JA-R performed the NO/Ozone analyses. All authors contributed to review and approval of the manuscript.

### Conflict of Interest

The authors declare that the research was conducted in the absence of any commercial or financial relationships that could be construed as a potential conflict of interest.
